# National alliance for Wilson’s disease: health policy in Costa Rica

**DOI:** 10.1186/s41124-016-0012-x

**Published:** 2016-07-25

**Authors:** Francisco Hevia-Urrutia, Ileana Alvarado-Echeverría, Alfredo Sanabria-Castro, Marta Sánchez-Molina, Luis Meza-Sierra, Alexander Parajeles-Vindas, Oscar Méndez-Blanca, Álvaro Sánchez-Siles, Manuel Saborío-Rocafort, Marcela Barguil-Gallardo, Iliana Chavarría-Quirós, Cecilia Monge-Bonilla

**Affiliations:** 1 Hospital San Juan de Dios, Caja Costarricense del Seguro Social, 4917-1000 San José, Costa Rica; 2 Hospital México, Caja Costarricense del Seguro Social, San José, Costa Rica; 3 Hospital Nacional de Niños, Caja Costarricense del Seguro Social, San José, Costa Rica

**Keywords:** Wilson’s disease, Ceruloplasmin, Hepatolenticular degeneration, Fulminant liver failure Costa Rica, National Alliance

## Abstract

Wilson’s disease is an inherited disorder in which defective biliary excretion of copper leads to its accumulation, particularly in the liver and brain. Mutations in the ATP7B gene on chromosome 13 cause Wilson’s disease. If left untreated it will cause liver failure, neurological damage, and will be life threatening. It is considered a rare disease afflicting approximately 1 in 30,000 persons worldwide, although this rate is similar in the different countries some places show higher incidence rates. Since Costa Rica reports the highest number of cases per population, essential public health initiatives that promote wellbeing, prevent disease complications, and prolong life among the affected population have been carried out during the last decades. The most recent lead in this matter is the conformation of the Costa Rica’s National Alliance for Wilson’s disease whose main objective is to provide practical, operational, timely and relevant guidance to patients, families, and healthcare professionals in the region for early diagnosis and treatment. The development and implementation of the National Alliance for Wilson’s disease activities is crucial because it will reaffirm that early intervention and appropriate treatment, will reduce if not eliminate the burden of Wilson’s disease.

## Background

Wilson’s disease (WD) also named hepatolenticular degeneration is a rare genetic disorder that causes excessive copper accumulation in the liver and brain and is fatal if not detected and treated [1]. The disease was named after Samuel Alexander Kinnier Wilson, M.D., who in 1912 reported pathologic findings of lenticular degeneration in the brain associated with cirrhosis of the liver [2]. The epidemiology of WD varies worldwide it is estimated that the pathology affects approximately 1 in 30,000 people [3] and one in 90–150 individuals carry a single abnormal Wilson’s disease gene. Countries like Costa Rica and Japan show the highest prevalence; 1 in 60 per million people; almost doubling world’s reports [4]; aspect that has become a health issue. The elevated number of cases in Costa Rica could be explained because: the high rates of consanguinity in the country, in which a small number of founding families date back to the eighteenth century [5] and low migration rates.

Despite the high incidence of WD in Costa Rica currently the relation between the number of deaths caused by hepatolenticular degeneration and the mortality by other liver diseases is not certain. Therefore a group of pioneers have been working together in the last years with the objective of studying profusely this disease and creating specific treatment and diagnosis guidelines for the region.

Axiom of Wilson’s disease in Costa Rica:

*“When approaching liver disease in Costa Rica; in patients under 30 years of age with chronic liver disease not secondary to alcohol consumption; the most likely diagnosis is Wilson’s disease.” Hevia-Urrutia M.D.*

### History of Wilson’s disease in Costa Rica

**Fig. 1:**
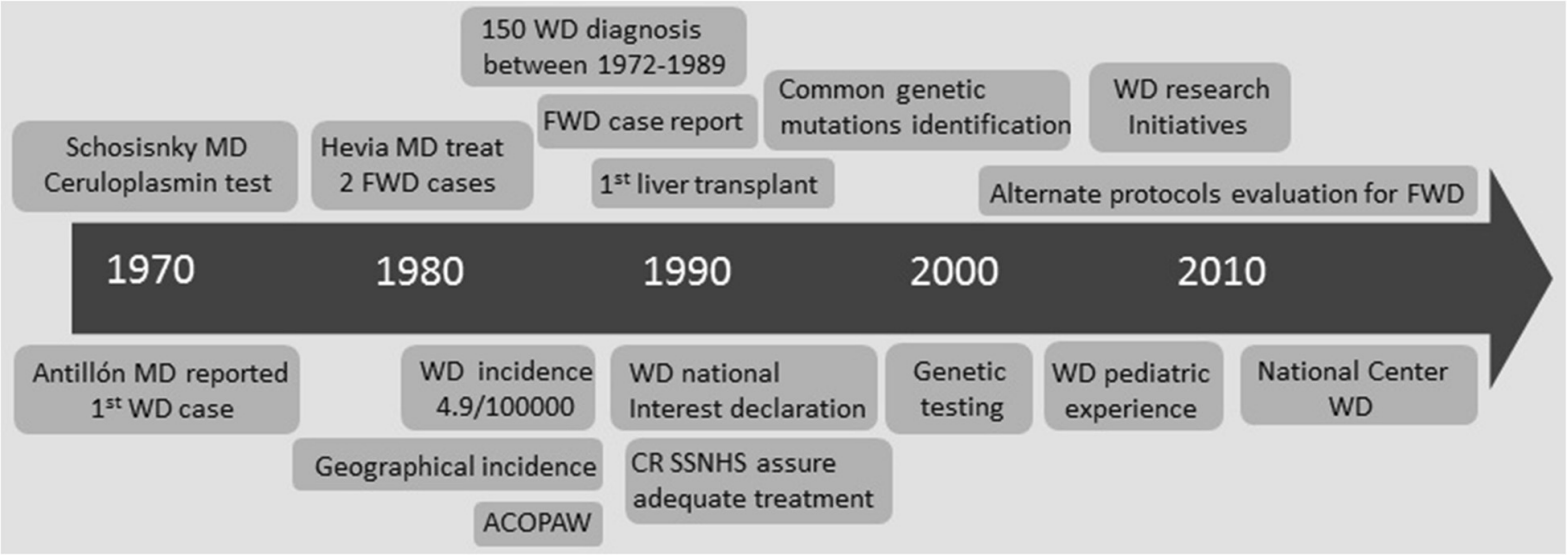
Timeline of Wilson’s Disease Events in Costa Rica. WD: Wilson’s disease, MD: Medical doctor, FWD: fulminant Wilson’s disease, ACOPAW: Costa Rican Wilson’s disease Patient Association, CR: Costa Rica, SSNHS: Social Security National Health System

**Fig. 2:**
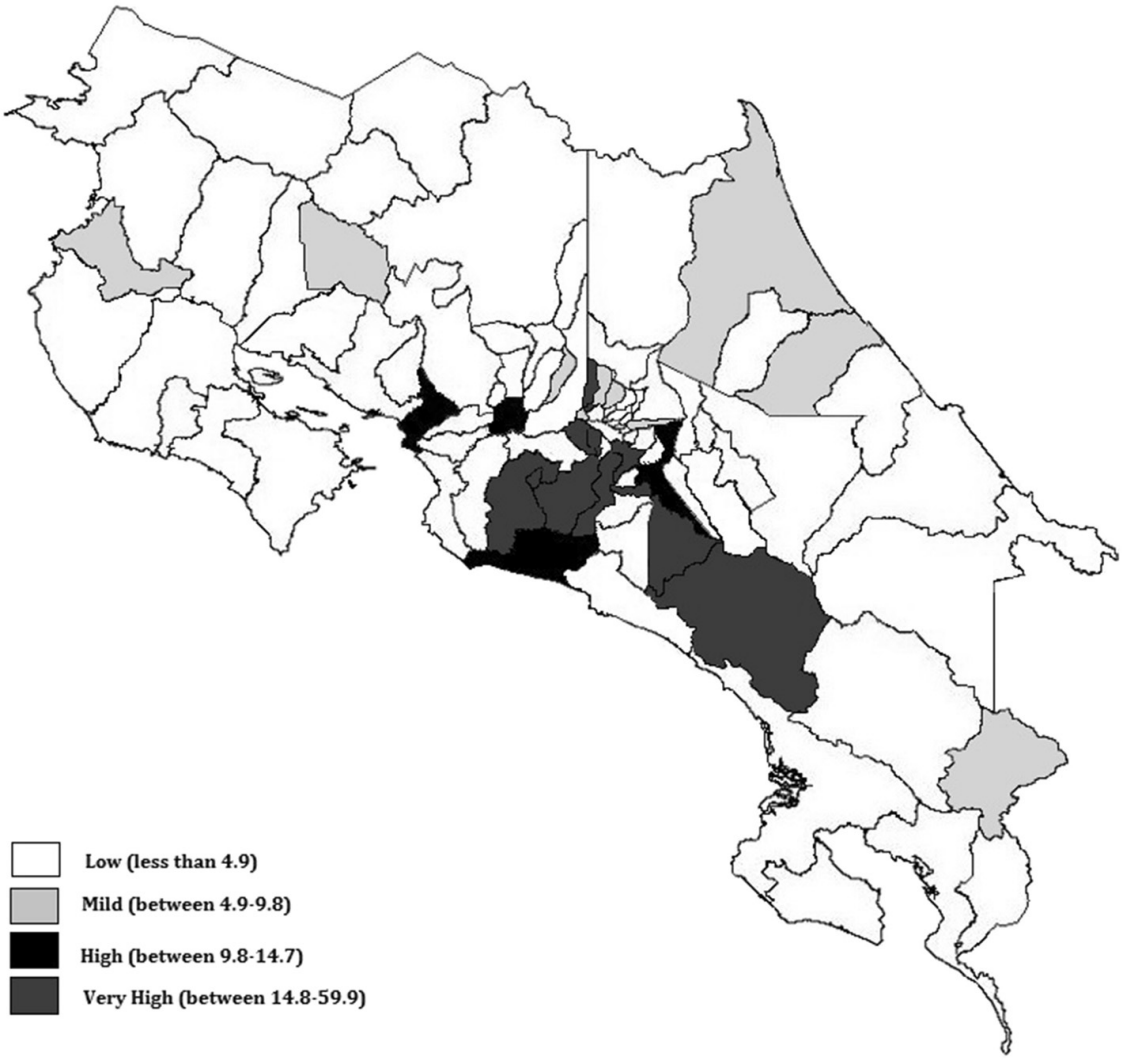
Incidence of Wilson’s disease in Costa Rica by canton from 1970 to 1989. Data is presented in cases per 100000 inhabitants

In 1970 Antillón-Salazar M.D. reported the first patient with WD in Costa Rica, a 17 year old man [6]. Around the same time, in Canada, Karl Schosinsky a Costa Rican M.D. contributed in the development of a precise, quantitative, enzymatic method for serum ceruloplasmin determination [7]. About thirteen years later Hevia-Urritia M.D., treated two young female patients (approximately 16 years old) with fulminant hepatic failure on the same month (Fig. 1), a rare condition at the time also described by McCullough et al. [8] In 1989, the incidence of WD in Costa Rica was reported as 4.9 per 100000 inhabitants, the highest in the world. Since the determination varies greatly upon location, Costa Rican regions with high incidence were identified and located in the central part of the country, showing close similarity to colonization cores (Fig. 2).

On 1990, Herra et al., reported that between 1972 and 1989, 150 cases of WD were diagnosed in Costa Rica; of these 120 were treated at Hospital San Juan de Dios. Seven patients died of acute liver failure, hemolytic anemia, encephalopathy, gastrointestinal bleeding or kidney insufficiency [9].

At the same moment, because of the high prevalence of WD in Costa Rica nationwide-inter-hospital working groups, consisting of medical microbiologists and pathologists, were established to analyze different diagnostic methods of copper metabolism for accuracy and effectiveness.

Hevia-Urrutia M.D and a group of patients founded in 1989 the Costa Rican Association of Patients with Wilson’s Disease (A.C.O.P.A.W.) [10]. The goal of this association is to bring WD patients and their families together to inform, educate, and discuss valuable information regarding medications, health care, research, family screening, and support. Since its early days, A.C.O.P.A.W has had an annual meeting the first week of October, led and coordinated entirely by WD patients.

A major achievement was also obtained in 1989, during Oscar Arias Sanchez’s government. The Costa Rican congress declared WD a “National Interest Disease”, this ensure the adequate treatment (penicillamine, zinc and trientine) and management of patients by the Costa Rican Social Security National Health System (CCSS) (Fig. 1).

During 1997 the determination of the specific genetic mutations for Costa Rican WD patients proved different from mutations in US patients and most parts of Europe [11]; however the mutations observed in these patients (AsnSer 1270) were the same as those found in patients with WD in Sicily, Italy [12, 13]. Since then, Wilson’s disease familial genetic mapping has been an important research line and has identify other genetic mutations that affect the population as well the most affected families in the country.

In 2009, the clinical presentation and demographic characteristics of the Costa Rican pediatric WD patients were described and showed similar characteristics to that in children diagnosed in other countries [14].

As worldwide in Costa Rica WD patients with fulminant hepatic failure are priority for liver transplant, nevertheless when this is not an option, innovative medical treatment with prostaglandins, vitamin E and haemoperfusion is administered [15]. In Costa Rica this management protocol has been used in the last 10 years and has shown positive outcomes in at least four patients (all women under 20 years of age) (Fig. 1).

In 2015 a collaborative alliance that includes gastroenterologists, researchers, neurologists, psychologists, psychiatrists, surgeons, geneticists, as well as other healthcare providers was created. The main purpose of this national alliance is to modify the way healthcare is delivered to WD patients, assuring that all Costa Rican hospitals diagnose, treat and manage WD accurately, effectively, rapidly and in a homogenous manner. The National Alliance for WD based at the San Juan de Dios Hospital includes healthcare professionals from other centers like the Hospital Mexico (hepatic transplant team) and the National Children’s Hospital (genetic screening).

In order to achieve its objectives the National Alliance for WD has establish a network that activates upon new possible WD diagnoses. Each member of this coalition has different roles but work in collaboration during the whole process which starts with diagnosis confirmation. Once the diagnosis is established members become fully involve in the family screening, best treatment option determination and disease management. The members also determine in conjunction priority research projects related to WD, and develop educational activities like workshops, exercise prescription and referral and other decision-making tools; addressed for healthcare professionals. These precise and effective mechanisms of interaction and communication have shown great results in approaching and solving urgent cases of WD, particularly in cases of fulminant hepatic failure.

The National Alliance for WD in Costa Rica aims to establish a Latin American Wilson’s disease network through the Latin American Hepatology Association (ALEH). This network will assist in the establishment of additional national alliances, centers and committees in other Latin American countries, to collaborate in the training of diagnostic, management and treatment protocols, along with providing educational and scientific support regarding WD.

Currently the National Alliance for WD in Costa Rica considers the possible establishment of collaboration with a research group in Pamplona, Spain; that has developed a successful gene therapy for WD in animal models. For the future the creation of joint collaborations and programs with other organizations outside Costa Rica that perform similar actions as the National Alliance for Wilson’s Disease are in scope. These institutions include: Medlineplus [16], National Library of Medicine Genetics Home Reference [17], NCBI Genes and Disease, Wilson’s Disease Association (WDA), American Association for the Study of Liver Diseases (AASLD), American Liver Foundation [18], European Society for Liver diseases (EASL), Canadian Liver Foundation (CLF) [19] and Euro Wilson Registry [20].

Because of the high prevalence of WD in Costa Rica current research is being conducted to improve diagnosis, management and treatment of WD patients. Some of the research initiatives include: new variations of D-penicillamine load tests to achieve earlier diagnosis, the significance of free copper levels in patients after diagnosis and during treatment, the use of medications such as tetrathiomolibdate in patients with neurological symptoms [21], long term results and impact of immediate physiotherapy in patients with neurological symptoms, programs for early detection of cognitive and psychological abnormalities during childhood in undiagnosed patients, WD screen training for school teachers, zinc as a possible treatment for pregnant patients and medical management of fulminant hepatitis secondary to WD with prostaglandins, haemoperfusion and vitamin E.

### Symptoms of Wilson’s disease

The liver is the first organ to be affected by the buildup of copper due to WD; however, patients are often misdiagnosed with infectious hepatitis. Excess copper can also produce psychiatric or neurologic symptoms [22, 23] which may cause the patient to present tremors, difficulty walking, talking and swallowing, along with various degrees of mental illness. Other signs and symptoms of WD include fatigue, lack of appetite, abdominal pain, jaundice, bruising, edema in the lower extremities, ascites and Kayser-Fleischer rings on ophthalmological examination [24]. In Costa Rica, the majority of WD patients exhibit liver disease and in more than 5% the pathology presents as fulminant hepatic failure. So it is crucial to test patients for WD if they have unexplained steatosis, liver failure, cirrhosis, cholestasis, and neurological impairment, specifically because of the high incidence of Wilson’s disease in the country.

### Specific guidelines for diagnosing Wilson’s disease

Early diagnosis and family screening of WD is fundamental, so treatment can be initiated before liver failure or neurological damage ensues. The 2001 International Meeting of Wilson’s disease, held in Leipzig, concluded that when evaluating a potential WD patient, these fundamental diagnostic elements should be included [25, 26]:

- Ceruloplasmin (ferroxidase enzyme) which is normally lower than 200 mg/L. When ceruloplasmin is low, deficiency disorders may occur, including neurological symptoms like Menkes disease [27]. Serum ceruloplasmin has also been related, since 1975, to the detection of problems during early gestation [28].
- Serum free copper, which is normally greater than 200 mcg/L.
- Hepatic copper, which is normally greater than 250 mcg/g dry weight.
- The presence of Kayser-Fleischer rings on slit-lamp examination. However rings may be absent in up to 50 % of patients with hepatic WD and most asymptomatic siblings, but present in other hepatic diseases such as primary biliary cirrhosis. In contrast, Kayser-Fleischer rings are present almost invariably in neurological WD [29] and in neurodegenerative diseases like Alzheimer’s disease [30].

While often the criteria of the Leipzig score are met [25], the combination of Kayser-Fleischer rings and a low serum ceruloplasmin (<0.1 g/L) level can be sufficient to establish a diagnosis of Wilson’s disease [26].

Nowadays, the genetic diagnosis is also a feasible option, especially for family screening. The sequence analysis of ATP7B gene is clinically available to identify the mutations in the ATP7B gene on chromosome 13. However, the existence of more than 500 mutants makes the genetic testing laborious and expensive [31] and in approximately 17 % of clinically confirmed WD cases, no mutation is identified [32]. Nevertheless significant genotype-phenotype correlation exists. ATP7B mutations result in absent or totally nonfunctional Wilson ATPase and are associated with severe hepatic disease [33, 34] (Table 1).

**Table 1:** Scoring system on Wilson’s disease [26]

Typical clinical symptoms and signs		Other tests	
Kayser-Fleischer Rings		Liver Copper (in the absence of cholestasis)	
Present	2	>5x ULN (>4 μmol/g)	2
Absent	0	0.8-4 μmol/g	1
Neurologic Symptoms^b^		Normal (<0.8 μmol/g)	-1
Severe	2	Rhodamine -positive granules^a^	1
Mild	1	Urinary copper (in absence of acute hepatitis)	
Absent	0	Normal	0
Serum Ceruloplasmin		1-2x ULN	1
Normal (>0.2 g/L)	0	>2x ULN	2
0.1-0.2 g/L	1	Normal but >5x ULN after D penicilamine	2
<0.1 g/L	2	Mutation analysis	
Coombs-negative hemolytic anemia		On both chromosomes detected	4
Present	1	On 1 chromosome detected	1
Absent	0	No mutations detected	0
TOTAL SCORE EVALUATION			
4 or more Diagnosis established			
3 Diagnosis possible, more tests needed			
2 or less Diagnosis very unlikely			

Scoring System on Wilson’s disease. 8th International Meeting on Wilson’s disease. Leipzig, 2001

^a^If no quantitative liver copper available, ^b^or typical abnormalities at brain magnetic resonance imaging. ULN, upper limit of normal

In Costa Rica, after a patient has been diagnosed it is essential that family members also get tested. The most successful WD detection and diagnostic method for family members of patients diagnosed with WD is as follows:

- Once a patient is identified with WD, their first and second degree relatives should undergo screening for WD through ceruloplasmin determination at the nephrology laboratory, which specializes in diagnosing copper metabolism abnormalities, including WD. Test results are available in a timely manner (less than 7 days).
- If ceruloplasmin level is low or normal-low, the result is reported to Hevia-Urrutia M.D. team and the individual will undergo 24-h urinary copper screening.
- In cases where both tests are abnormal or positive for WD, further diagnostic procedures such as a 24-h D-penicillamine challenge, liver biopsy, and genetic testing may take place.

### Guidelines for treatment of Wilson’s disease

Patients in Costa Rica diagnosed with WD (symptomatic and asymptomatic) are referred to a tertiary care level hospital and followed by specialists in gastroenterology and hepatology. Patients diagnosed with fulminant WD undergo an inter-hospital protocol for treatment of fulminant hepatic failure and liver transplant.

Several drugs are available for the treatment of Wilson’s disease, including D-penicillamine, trientine, zinc, tetrathiomolybdate, and dimercaprol. These drugs are designed to remove excess copper and to prevent its accumulation. If treatment is initiated immediately after diagnosis, for both presymptomatic and symptomatic patients, deterioration can be avoided and life expectancy can be comparable to healthy subjects; of course, a patient must be compliant with their therapy. In Costa Rica, treatment with zinc during pregnancy and nursing is considered because of the teratogenic effects of chelating agents like D-penicillamine and trientine.

Diet is an additional consideration and WD patients should avoid foods naturally high in copper including: chocolate, nuts, mushrooms, crustaceans, soy and gelatin, along with the use of cooking utensils containing copper. To ensure the safety of water flowing through copper pipes, water should run for a few minutes before drinking [29]. The fact that in Costa Rica rural areas, from where many WD patients come from, have a high copper diet constitutes an aspect that must be taken into account (Table 2).

**Table 2:** Treatment recommendations for Wilson’s disease [26]

• Initial treatment for symptomatic patients with Wilson’s disease should include a chelating agent (D-penicillamine or trientine). Trientine may be better tolerated	• Patients with acute liver failure due to Wilson’s disease should be treated with liver transplantation when the revised King’s score is 11 or higher
GRADE II-1, B, 1	GRADE II-2, B, 1
AASLD Class I, Level B	AASLD Class I, Level B
• Zinc may have a role as a line therapy in neurological patients	• Patients with decompensated cirrhosis, unresponsive to chelation treatment, should be evaluated promptly for liver transplantation
GRADE II-2, C, 2	GRADE II-2, B, 1
AASLD Class II, Level C	AASLD Class I, Level B
• Treatment of presymptomatic patients or those with neurological disease on maintenance therapy can be accomplished with a chelating agent or with zinc	• Treatment for Wilson’s disease should be continued during pregnancy, but dosage reduction is advisable for D-penicillamine and trientine
GRADE II-1, B, 1	GRADE II-3, B, 1
AASLD Class I, Level B	AASLD Class I, Level C
• Treatment is lifelong and should not be discontinued, unless liver transplantation is performed	• For routine monitoring, serum copper and ceruloplasmin, liver enzymes and international normalized ratio, functional parameters, complete blood count and urine analysis as well as physical and neurological examinations should be performed regularly, at least twice annually
GRADE II-1, B, 1	GRADE II-2, B, 1
AASLD Class I, Level B	AASLD Class I, Level C
• If zinc is used, careful monitoring of transaminases is needed, with changing to chelators if these laboratory parameters are increasing	• The 24-h urinary copper excretion on medication and after 2 days of cessation of therapy should be measured at least yearly. The estimated serum non ceruloplasmin bound copper may be another useful parameter to control therapy
GRADE C1	
AASLD Class I, Level B	
• Patients should avoid intake of foods and water with high concentrations of copper, especially during the year of treatment	
GRADE II-3, B, 2	GRADE II-3, B, 1
AASLD Class I, Level C	AASLD Class I, Level C

8th International Meeting on Wilson’s disease. Leipzig, 2001

## Conclusion

While hepatolenticular degeneration is a rare disease worldwide, it is much more common in Costa Rica. Important advances relating the pathology have being made in the last decades through a series of actions and efforts in health policy, politics, and multidisciplinary work. One of the latest interventions is the foundation in 2015 of the National Alliance for Wilson’s disease.

The ultimate goal of the Costa Rica’s National Alliance for Wilson’s disease is to promote awareness and early intervention of WD in a country un-proportionally afflicted with this deadly disease. Currently this initiative has begun implementing actions by evaluating guidelines for early diagnosis and adequate treatments, dissemination of relevant information on current research, legal issues and the support for WD patients and families; that have proof substantial reduction on Wilson’s disease affliction.

The future prospects of the Costa Rican WD National Alliance is the establishment of international clinical and research collaborations with other Latin American countries, Europe and Japan. As well the development of relations with similar organizations like the Wilson Disease Centers of Excellence. These mutual benefit collaborative initiatives are the cornerstone for future diagnosis, treatment and management of WD.

## Abbreviations

AASLD, American Association for the Study of Liver Diseases; ACOPAW, Costa Rican Wilson’s Disease Patient Association; ALEH, Latin American Hepatology Association; CCSS, Costa Rican Social Security National Health System; EASL, European Society for Liver Diseases; CLF, Canadian Liver Foundation; WD, Wilson’s disease; WDA, Wilson’s Disease Association
